# Supplementary material for: Pathway analysis in metabolomics: Recommendations for the use of over-representation analysis
Source: PLoS Comput Biol. 2021 Sep 7;17(9):e1009105. doi: 10.1371/journal.pcbi.1009105 (PMC8448349; doi:10.1371/journal.pcbi.1009105)
Supplement: S1 Supporting Information — Fig A: The effect of the number of input metabolites on the number of significant pathways (p ≤ 0.1) across all datasets. All metabolites in the dataset were ranked by their raw p-value which was calculated using t-tests to determine the level of differential abundance between two study groups. Beginning with the compound with the lowest p-value, the list of DA metabolites was created by adding one compound at a time (x-axis). ORA was performed using this list and the number of significant pathways at p ≤ 0.1 is shown on the y-axis. Bonferroni adjusted p-value thresholds are indicated using red markers and BH-FDR adjusted q-values are indicated using black markers. Table A: Significant pathways (P ≤ 0.1) obtained with KEGG, HumanCyc, and Reactome using the Yachida et al. dataset. Pathways with similar biological function significant at P ≤ 0.1 using at least two pathway databases are highlighted in bold. Fig B: Overlap coefficient values between all metabolites in significant pathways (p ≤ 0.1) detected using KEGG and Reactome. Fig C: Metabolite misidentification. Heatmaps showing pathway loss rate and pathway gain rate at varying percentages of metabolite misidentification by (a) identical chemical formula and (b) molecular mass within a +/- 20ppm window. Colour bar corresponds to pathway loss/gain rate, with darker colours representing lower rates. Misidentification by chemical formula shown up to 5%, whereas misidentification by mass shown up to 6%, as these are the highest values calculatable (based on limited replacement compounds) across all datasets. (DOCX) [file pcbi.1009105.s001.docx]

**S1 Supporting information**: Pathway analysis in metabolomics: recommendations for the use of over-representation analysis

## Authors:

**Cecilia Wieder ^1^, Clément Frainay ^2^, Nathalie Poupin ^2^, Pablo Rodríguez-Mier ^2^, Florence Vinson ^2^, Juliette Cooke ^2^, Rachel PJ Lai ^3^, Jacob G Bundy ^4^, Fabien Jourdan ^2, 5^, Timothy Ebbels* ^1^**

^1^ Section of Bioinformatics, Division of Systems Medicine, Department of Metabolism, Digestion, and Reproduction, Faculty of Medicine, Imperial College London, London, UK

^2^ Toxalim (Research Centre in Food Toxicology), Université de Toulouse, INRAE, ENVT, INP-Purpan, UPS, Toulouse, France

^3^ Department of Infectious Disease, Faculty of Medicine, Imperial College London, London, UK

^4^ Section of Biomolecular Medicine, Division of Systems Medicine, Department of Metabolism, Digestion, and Reproduction, Faculty of Medicine, Imperial College London, London, UK

^5^ MetaToul-MetaboHUB, National Infrastructure of Metabolomics and Fluxomics, Toulouse, France.

*t.ebbels@imperial.ac.uk


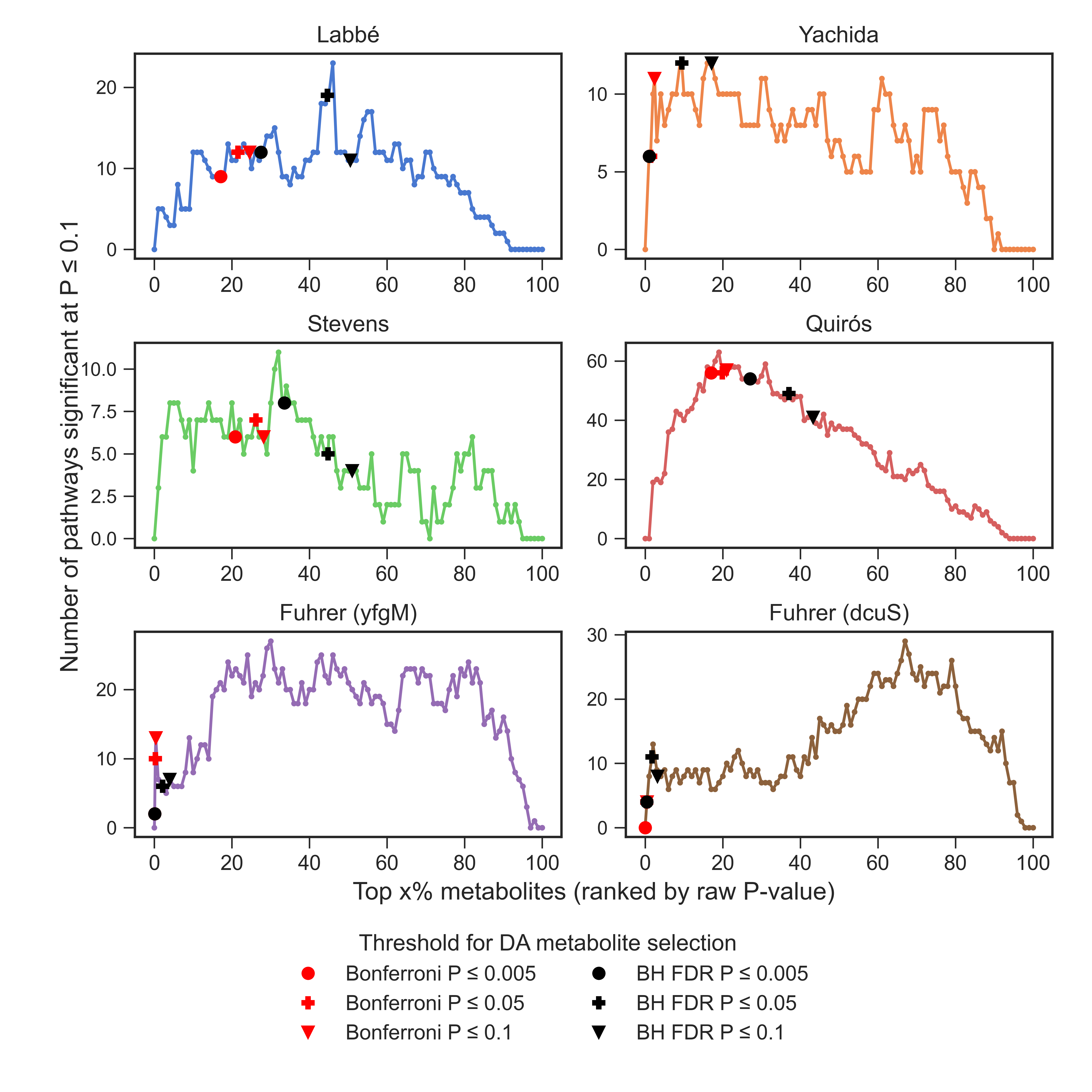


Fig A: **The effect of the number of input metabolites on the number of significant pathways (p ≤ 0.1) across all datasets.** All metabolites in the dataset were ranked by their raw p-value which was calculated using t-tests to determine the level of differential abundance between two study groups. Beginning with the compound with the lowest p-value, the list of DA metabolites was created by adding one compound at a time (x-axis). ORA was performed using this list and the number of significant pathways at p ≤ 0.1 is shown on the y-axis. Bonferroni adjusted p-value thresholds are indicated using red markers and BH-FDR adjusted q-values are indicated using black markers.

Table A: **Significant pathways (P ≤ 0.1) obtained with KEGG, HumanCyc, and Reactome using the Yachida et al. dataset**. Pathways with similar biological function significant at P ≤ 0.1 using at least two pathway databases are highlighted in bold.

| KEGG | HumanCyc | Reactome |
| --- | --- | --- |
| Ubiquinone and other terpenoid-quinone biosynthesis – Homo sapiens (human)  Valine, leucine and isoleucine degradation – Homo sapiens (human)  Valine, leucine and isoleucine  biosynthesis – Homo sapiens (human)  **Tyrosine metabolism – Homo sapiens (human)**  **Phenylalanine, tyrosine and tryptophan biosynthesis – Homo sapiens (human)**  Glyoxylate and dicarboxylate metabolism – Homo sapiens (human)  **Aminoacyl-tRNA biosynthesis – Homo sapiens (human)**  2-Oxocarboxylic acid metabolism – Homo sapiens (human)  Biosynthesis of amino acids – Homo sapiens (human)  Biosynthesis of cofactors – Homo sapiens (human)  Melanogenesis – Homo sapiens (human)  Shigellosis – Homo sapiens (human)  Central carbon metabolism in cancer – Homo sapiens (human) | L-serine degradation  L-phenylalanine degradation  CDP-diacylglycerol biosynthesis  Ceramide de novo biosynthesis  Hydrogen sulfide biosynthesis II (mammalian)  **L-tyrosine biosynthesis**  Phosphatidylserine biosynthesis II  L-cysteine biosynthesis III (from L-homocysteine)  **Diacylglycerol and triacylglycerol biosynthesis**  **tRNA charging**  L-dopa and L-dopachrome biosynthesis | PAOs oxidise polyamines to amines  Hydrolysis of LPE  Triglyceride catabolism  Signaling by VEGF  Metabolism of nitric oxide: NOS3  activation and regulation  eNOS activation  Nitric oxide stimulates guanylate cyclase  Platelet homeostasis  VEGFA-VEGFR2 Pathway  VEGFR2 mediated vascular permeability  Metal sequestration by antimicrobial proteins  **Triglyceride biosynthesis**  **Triglyceride metabolism**  Iron uptake and transport |


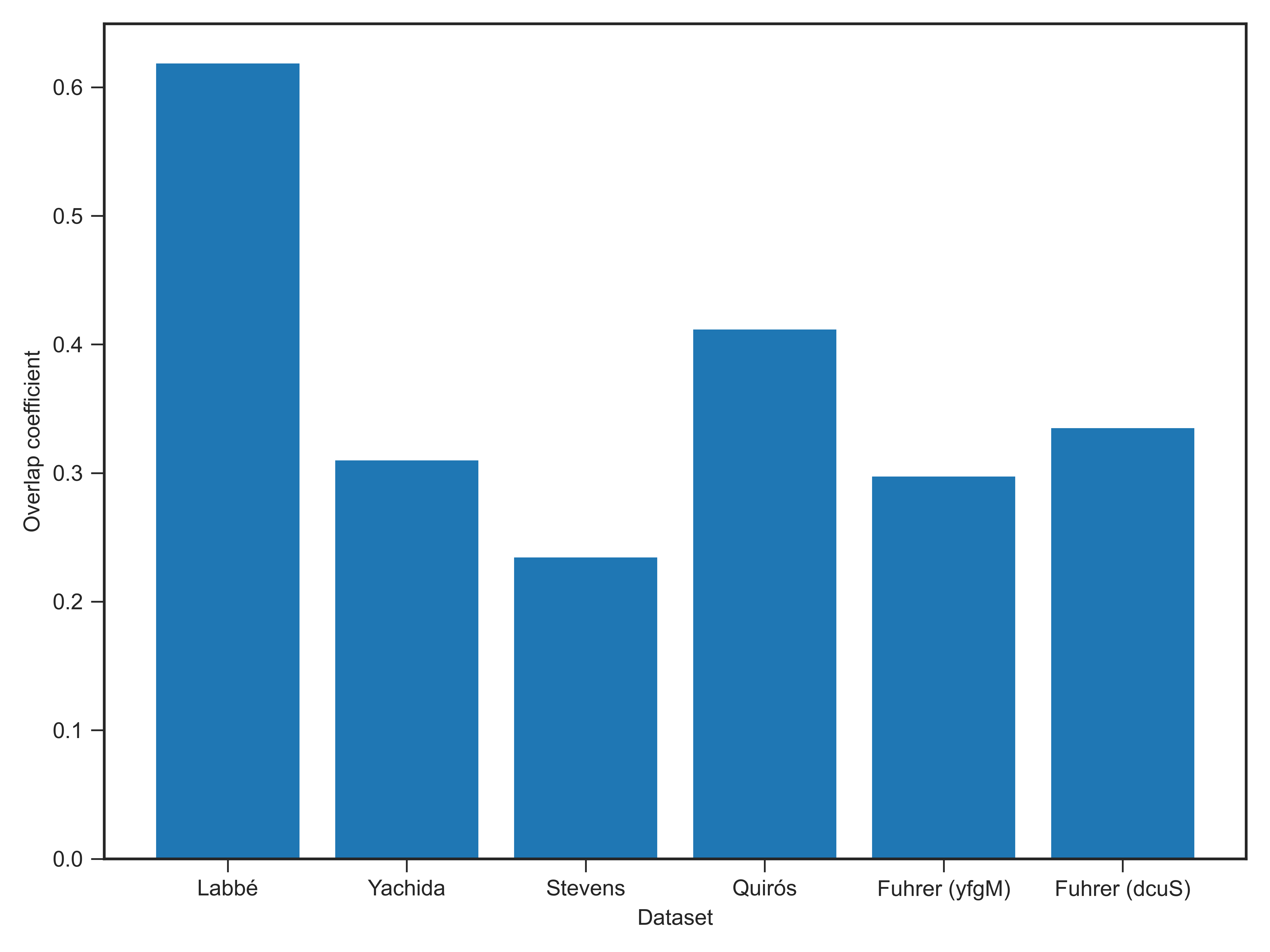


Fig B: **Overlap coefficient values between all metabolites in significant pathways (p ≤ 0.1) detected using KEGG and Reactome.**


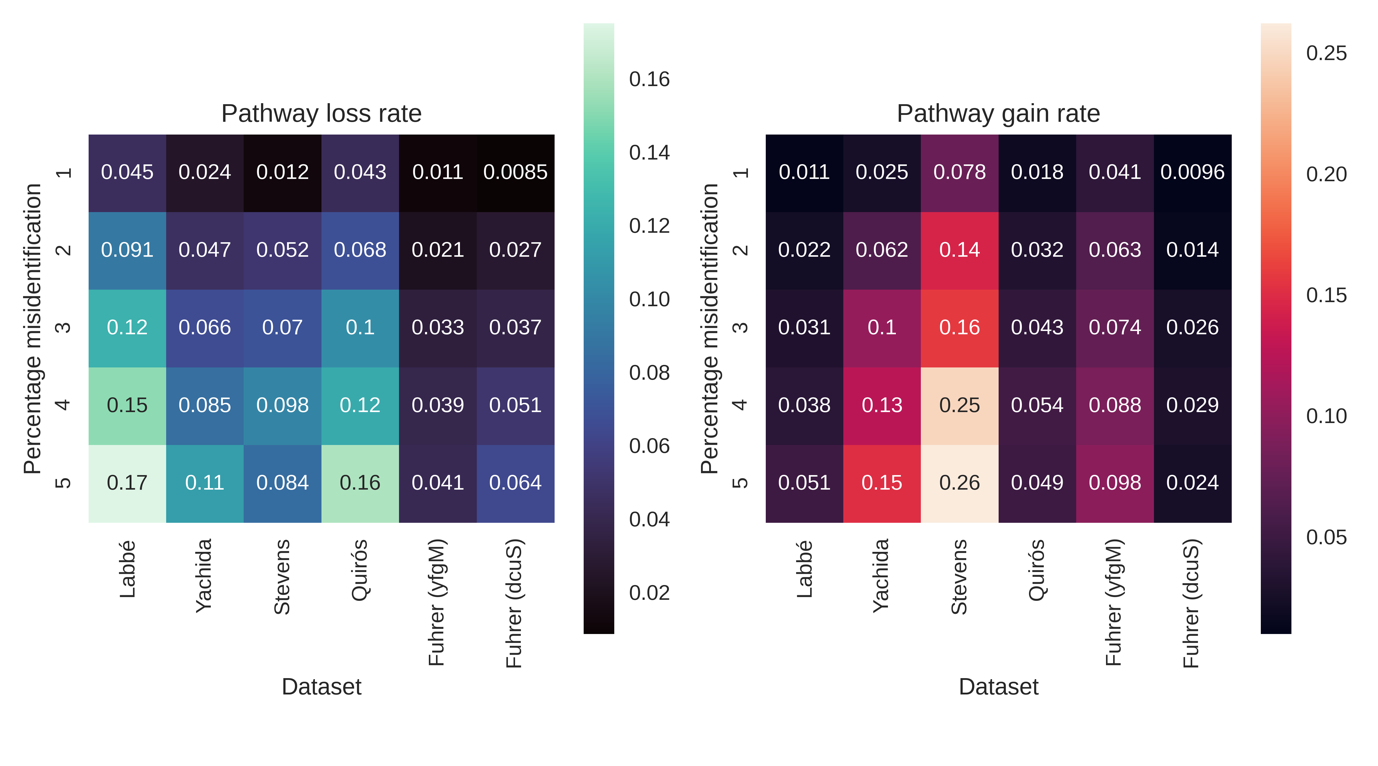

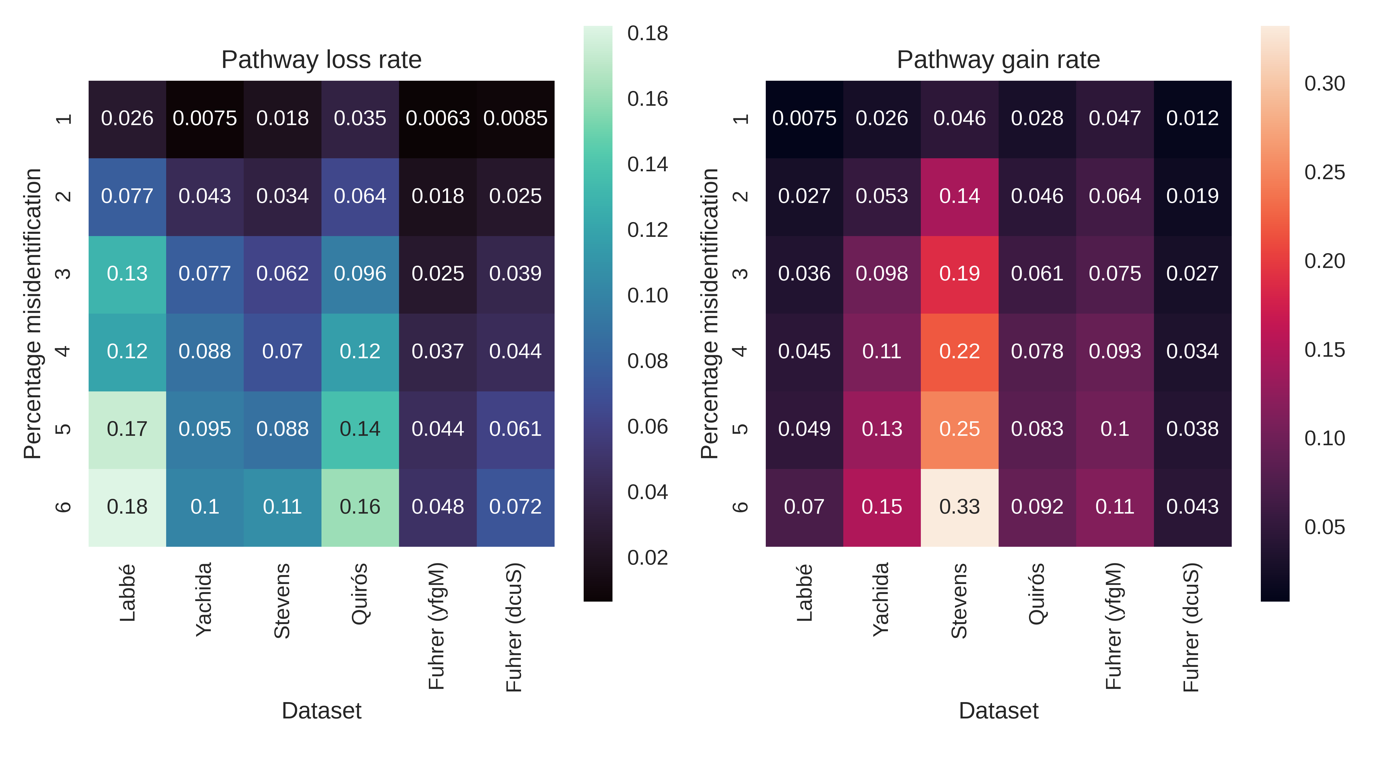


**a**

**b**

Fig C: **Metabolite misidentification**. Heatmaps showing pathway loss rate and pathway gain rate at varying percentages of metabolite misidentification by (a) identical chemical formula and (b) molecular mass within a +- 20ppm window. Colour bar corresponds to pathway loss/gain rate, with darker colours representing lower rates. Misidentification by chemical formula shown up to 5%, whereas misidentification by mass shown up to 6%, as these are the highest values calculatable (based on limited replacement compounds) across all datasets.
