# Supplementary material for: Pathway analysis in metabolomics: Recommendations for the use of over-representation analysis
Source: PLoS Comput Biol. 2021 Sep 7;17(9):e1009105. doi: 10.1371/journal.pcbi.1009105 (PMC8448349; doi:10.1371/journal.pcbi.1009105)
Supplement: Attachment — Submitted filename: Response_to_reviewers_v1.pdf [file pcbi.1009105.s002.pdf]

Section of Bioinformatics
Division of Systems Medicine
Department of Metabolism, Digestion and Reproduction
Imperial College London

Office 131 Sir Alexander Fleming Building
Exhibition Road
South Kensington
London SW7 2AZ
Tel: +44 (0)20 7594 3160

t.ebbels@imperial.ac.uk
http://www.imperial.ac.uk/people/t.ebbels

28 July 2021

Dr. Tim Ebbels BA PhD FRSC
Reader in Computational Bioinformatics

Dear Editor,

Thank you for your email and for the reviewers' comments concerning our manuscript entitled "Pathway analysis in metabolomics: pitfalls and best practice for the use of over-representation analysis". We sincerely appreciate all the valuable comments and suggestions that have been made which have helped us to improve the quality of this manuscript.

We have carefully studied each of the comments from the reviewers and have used these to improve our manuscript. Our responses to the reviewer comments and concerns are outlined below in a point-by-point manner, highlighted in blue font.

We look forward to hearing from you regarding our submission and to respond to any further questions and comments you may have.

Yours sincerely,

Timothy Ebbels
Imperial College London

Please note that all line numbers indicated in this response document correspond to the **tracked changes** version of the manuscript, rather than the clean copy of the manuscript. This is due to a known issue with MS Word where the line numbers on a document with tracked changes can be inconsistent.

Reviewer 1

Reviewer #1: Wieder et al examine how different parameters for pathway over-representation analysis (ORA) influence the results of metabolomics data analysis. They use five experimental metabolomics data sets (from humans, mouse and E. coli) to test the relationship between ORA parameters and ORA results. The study is relevant for the field because it nicely illustrates the strong influence of ORA parameters on the outcome of the analysis. However, my main concern is that the authors cannot identify the best parameter configuration because they lack a proper reference of what is true (they mention this also in the discussion). Specific comments are below:

We are glad to hear that the reviewer finds the study relevant and important. We would like to highlight that, while a ground-truth dataset would be valuable, it is not the aim of this paper to evaluate the accuracy of ORA. Instead we aim to demonstrate pitfalls relevant to practical application of the approach.

1) The authors claim in the abstract that they used in-silico simulations, thus I expected that they simulated metabolic changes (e.g. with a dynamic model) and used ORA to recover the true in silico perturbation. Instead they use real data, which is great, but it is difficult to judge which parameter configuration is best. The authors mention themselves that the study lacks a "ground-truth dataset". The authors should at least better describe the nature of such a ground-truth dataset/ result. They should also describe better what they mean with in silico simulation.

We thank the reviewer for this comment, and agree that more description of a ground-truth dataset would be useful. As such, we have elaborated on this in the discussion, to discuss the various possible sources of ground truth data, along with their strengths and weaknesses:

L721-731: "This study is limited by the lack of availability of a ground-truth dataset where the identities of enriched pathways are known. Possible sources of ground-truth data include simulations based on genome-scale metabolic models, in which enzymes in specific pathways are knocked out or the flux through reactions altered. Alternatively, one could insert artificial pathway signals into simulated or real data by altering the relative abundance levels of metabolites involved in the target pathways. Experimental datasets such as gene knockouts or knock-downs offer more realistic forms of ground truth datasets, which more accurately reflect the complexity of a biological system. Both simulated and experimental ground-truth datasets have limitations, however, such as the former being too simplistic, or the inability to pinpoint the exact pathway(s) affected by a perturbation in the latter. Nevertheless, such datasets might enable quantification of a wider variety of performance metrics than available here."

We agree that the word "simulation" in the abstract may give an expectation of the use of in silico techniques, such as genome-scale models. We have therefore removed the reference to

in-silico simulations in the abstract. By simulation based on experimental data, we aim to convey that we are manipulating the original data to reflect changes such as misidentification of metabolites, reducing background list size, modifying the list of differential metabolites, or simulating different assay types by selecting compounds based on polarity.

2) The authors selected 5 experimental data sets for their study. They could better describe in the main text (instead of Table 1) why they selected these data and which conditions/organisms are investigated. For example, why did they select (only) two strains out of 3800 E. coli strains in Fuhrer et al? In fact, these data contain some information about the "ground truth", because in many cases the deleted gene can be assigned to a metabolic pathway.

We thank the reviewer for this excellent point. We have revised the first paragraph of the Results to provide more rationale for the dataset selection and direct the interested reader to Table 1 of the methods for more details.

L203-207: "First, we examined several factors which are common to all ORA applications, beginning with the background set. Five publicly available metabolomics datasets have been used throughout this work (Table 1, see Methods). These datasets, obtained using untargeted mass-spectrometry (MS), were selected to encompass a diverse range of organisms, sample sources, and experimental conditions."

We have decided to keep the main paragraph describing the datasets in terms of sample source, size, etc. and Table 1 which provides detailed information on each dataset in the methods section, as we view this more as part of the materials and methods than a result.

We have further added a few lines to the methods section to highlight our selection process for these datasets:

L784-787: "Five publicly available untargeted metabolomics datasets were used in this work (Table 1). The aim of this work was to select a small sample of typical metabolomics studies to illustrate the effects of changing ORA parameters. The inclusion criteria for a dataset were: i) it should be publicly available, ii) it should contain over 100 annotated metabolites, and iii) there should be at least two study groups."

With regards to the Fuhrer et al. dataset, we aimed to select only two strains as good examples to demonstrate the behaviour of ORA, but also to avoid over complicating the paper with a large number of examples. These two strains were chosen because out of >3,800 strains they were amongst those with the highest numbers of significant pathways obtained using ORA. A large effect size in unaltered data (i.e., without misidentifying the data or reducing the background set size for example) makes it easier to demonstrate how different parameters can affect ORA results.

We agree that there is an element of ground truth to these datasets with respect to the gene knockouts, but there are also a number of limitations associated with the ablation of genes, such as difficulty pinpointing the perturbed pathway, long range effects or other genes

compensating for the original gene's function. Furthermore, as this dataset did not use any separation step prior to mass spectrometry, there is a much higher degree of uncertainty surrounding the metabolite identifications than in the other datasets. Hence we believe that using this data as the sole source of ground truth would give a biased assessment picture of the accuracy of ORA.

3) A main concern is the selection of Databases. Obviously, the best choice is a genome-scale reconstruction of metabolism of the respective organism and I wonder why the authors did not consider them; at least for the E. coli data, mouse and HeLa cells.

We appreciate the reviewer's insightful suggestion on the use of genome-scale metabolic models. While we do not question the relevance or validity of genome-scale reconstructions of organism metabolism, we did not use them in this work as we aimed to demonstrate the key pitfalls in a typical metabolomics pathway analysis workflow (although not necessarily the ideal workflow), and therefore selected commonly used pathway databases such as KEGG. For most practitioners, the simplest way to obtain sets of metabolic pathways involves the use of pathway databases. As such, we hope that the use of conventional pathway sets will make this work more accessible to the wider metabolomics community who may not have the knowledge or tools to run genome-scale metabolic models.

We do acknowledge it is possible to extract pathway sets from genome-scale network models, but these may not be as comprehensive as the KEGG pathways, for example. Furthermore, artificial reactions are often added to these networks for the purpose of flux modelling and are combined into unspecific or miscellaneous pathways which tend to be large and may therefore bias the pathway analysis. Additionally, although genome-scale metabolic models provide detailed information about a metabolic system, such as mass- and energy-balanced reactions, such information cannot be directly used in ORA. Genome-scale models also contain information about the cellular compartment of reactions (at least in eukaryotes), but in most metabolomics studies this information is not readily accessible, which increases the complexity of mapping metabolites onto such networks. Finally, all the analyses we have conducted relating to the pitfalls/issues we have examined would remain the same regardless of the source of the pathways.

4) The authors could give a better overview about the parameters tested and better quantify their relevance relative to each other. The recommendations in the discussion are not specific enough. For example, how could one derive a "consensus" pathway signature.

We thank the reviewer for highlighting the difficulty around deriving "consensus" pathway signatures.

We have added the following to the manuscript which aims to quantify the level of metabolite consensus in the significant pathways detected using KEGG and Reactome databases:

L358-362: "To quantify this effect, we pooled all metabolites from the significant pathways ($p \leq 0.1$) detected using KEGG and Reactome and calculated the OC between the two sets of compounds for each dataset. OC values ranged from 0.23 (Stevens dataset) to 0.62 (Labbé

dataset) (Fig S2), indicating low to medium consensus between ORA based on different pathway databases.”

- *The overlap coefficient (OC) is described in the same section as well as in the methods.*

Fig S2:

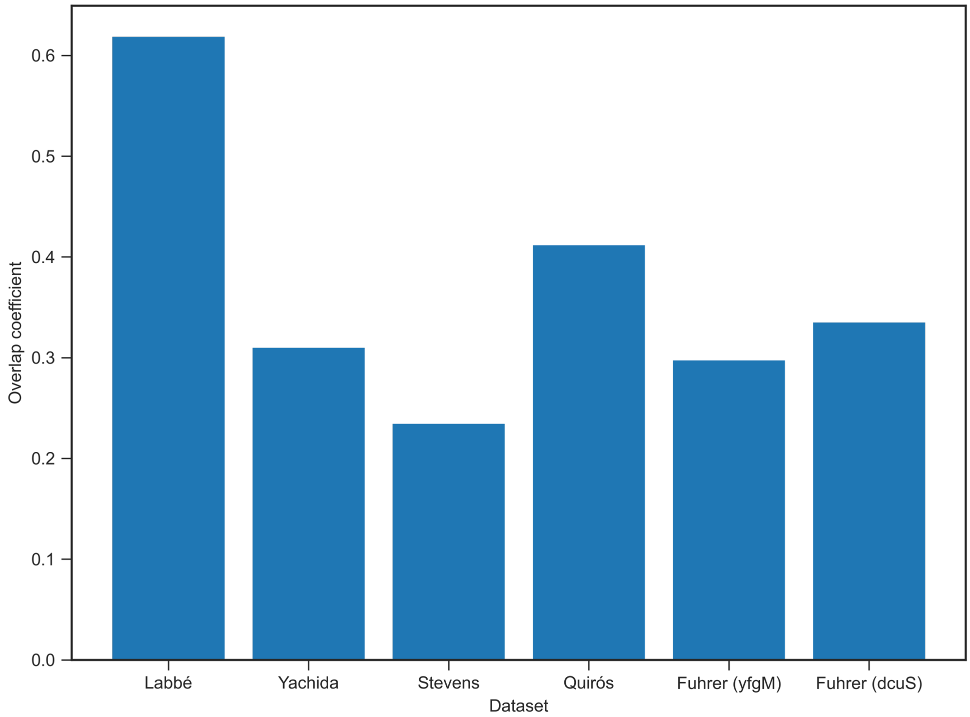

Finding the best method for deriving a consensus signature is a substantial task that we believe to be out of the current scope – especially without ground truth data - but is an important area for future research. However, as practical advice, we have modified the main text to emphasise the use of biochemical knowledge:

L706-712: “A general recommendation is to use multiple pathway databases and derive a consensus signature across these, if possible, reinforced by current knowledge of the underlying biochemistry of the system investigated. The use of integrative databases encompassing several pathway databases, such as the ConsensusPathDB [36], or interactive tools to simultaneously visualise pathways from different databases such as PathMe [37] may be beneficial and reflect ongoing efforts to harmonise pathway resources.”

Reviewer 2

Reviewer #2: The authors assess parameters used in pathway enrichment analysis using 5 publicly available MS-based metabolomics datasets. While those dealing with these tools have surely identified inconsistencies in results according to the tools and parameters used, the exercise of testing the boundaries and consequences in results of mis-use of the tools is interestingly quantified by the authors. In addition it is of value the section on recommendations on the best practice to use over-representation analysis (ORA) in the metabolomics field.

The manuscript is well-written but requires improvement in certain sections.

Title/introduction – It is worth mentioning that ORA is also known as metabolite enrichment analysis, that might even be a more common name used within the metabolomics community.

We thank the reviewer for pointing this out. We have modified this line to the introduction to reflect this:
L92-93: "ORA (referred to by some authors as metabolite enrichment analysis) has found widespread use in the identification of significantly impacted pathways in numerous metabolomics studies [9–13].".

We have decided not to include metabolite enrichment analysis in the title as we wish to keep it specific to ORA and avoid confusion with other pathway analysis methods such as gene set enrichment analysis or Goeman's globaltest applied to metabolomics data, which some also refer to as metabolite enrichment analysis.

Methods

L501 – for dataset MTBLS135 the text mentions that the sample type is plasma, while Table 1 mentions tissue. So here it is important to rectify and harmonize. In addition, the files of the uploaded dataset mention 'serum' and not plasma. This might sound like a detail, but one should be precise, as the two sample types (serum and plasma) are not interchangeable.

We thank the reviewer for identifying this inconsistency and can confirm that the data used for MTBLS135 was indeed the tissue dataset rather than the serum dataset. This has been rectified in L501 of the text to reflect the same sample source as in Table 1.

L502 – dataset MTBLS136: I could not retrieve any data files in the Metabolights repository for this study! Supplementary Materials of the associated publication do not contain the metabolomics data itself per sample. So I could also not confirm the number of samples (controls and estrogen-users).

We thank the reviewer for looking into the dataset and the number of samples. We can confirm that as of 9/07/21 the raw and processed data for MTBLS136 are available at https://www.ebi.ac.uk/metabolights/MTBLS136/files. Please note that due to the file size of the raw data, the files on the page may take some time to load.

L506 – as for all the other datasets, it is important to mention the number of samples for the last dataset Fuhrer et al. And was the negative mode subdataset used or the positive mode or both? In the results, it is then mentioned 2 subsets from this particular study, so this needs to be clarified in the Methods.

We thank the reviewer for highlighting the need for this clarification. We can confirm that each of the single gene knockout E. coli strains used in the Fuhrer et al. study had three biological replicates. We combined data from both the positive and negative ionisation modes to use in this study. The text has been modified to reflect this:

L794-799: "The final dataset is available from EBI BioStudies (S-BSST5) and consists of >3,800 single-gene E. coli knockouts each with 3 biological replicates [44]. Data from the positive and negative ionisation modes was combined to provide the final matrix of putative compound identifications and relative abundances for each. We selected two knockout strains to investigate from this dataset which were amongst those with the highest effect size (based on the number of significant pathways detected using ORA): ΔyfgM and ΔdcuS."

Table 1 – where does the total number of metabolites mapping to KEGG compounds was extracted from? Analysis within this manuscript or extracted from the original datasets?

In the Labbé et al., Yachida et al., Quirós et al., and Fuhrer et al. datasets the metabolite to KEGG compound mapping was provided by the original authors and can be found in the supplementary information or metabolite abundance matrix for each dataset. The Stevens et al. dataset was generated by the original authors using the Metabolon platform and several metabolite identifiers for each compound are provided including HMDB IDs. The HMDB IDs were mapped to KEGG IDs using the MetaboAnalyst ID conversion tool. The metabolite identifier mappings are described in section 1.3, L837.

L525 – metabolite ID conversion
This is a stress point of identification and according to the algorithms used, it can over-identify and thus overestimate metabolite coverage or if too conservative, it can assign only a part of possible metabolites.
For example: when one measures an amino acid, will it be immediately assigned to L-amino acid? An amino acid can be also D-amino acid in a biological environment, however this type of assignment is hardly assessed and possibly not even feasible to know using regular LC/CE-MS techniques (one would need to use chiral chromatography fo example). And in the likely even of not knowing, will it be assigned to D/L-amino acid or assumed to be L-amino acid?
Another example are acids and salts and ions (for example: glutamic acid vs glutamate vs sodium glutamate (or any other salt)): will these be assigned to the same metabolite ID or to different ones?
As different metabolite ID convertors (tool in MetaboAnalyst, too in BioCyc, etc) were used, it is likely that these will produce different results!! This aspect deserves some explanation and words of caution in the manuscript. Will the IDs be back-converted to the same list of IDs when using convertors from other databases? This would be good to check.

We thank the reviewer for highlighting this important point. We would like to clarify that the metabolite identification in each of the datasets used in this study was performed by the original authors and upstream of the analysis in this work. In each dataset, the compounds were already mapped to either KEGG or HMDB IDs, which are specific enough to contain information about the stereochemistry of the amino acids for example (e.g. L- or D- amino acids). As metabolite identification not in the scope of this work, we used the identifications provided by the authors.

In terms of acids and salts, the reviewer is correct in assuming that there can be a discrepancy between the mapping of these compounds. For example, in KEGG, L-glutamate and glutamic acid both map to the same identifier, C00025. In the ChEBI database (which is

used by Reactome), these map to two separate compound entries. This can indeed be an issue as it is not guaranteed that the acid and salt form of a compound will be mapped to the same pathways. In cases like these, the conversion tools rely on the cross-references between databases (for example L-glutamate/glutamic acid in KEGG will map to L-glutamic acid in ChEBI and vice versa).

While we do appreciate that mapping compounds between different pathway databases remains a bottleneck in the metabolomics pathway analysis workflow, we believe we have selected the most appropriate tools (and in some cases such as BioCyc - the only tool) that will convert IDs between databases in an automated manner.

The reviewer's suggestion to check back conversion of identifiers is a good one. Taking the Labbé dataset as an example, it contains 267 metabolites mapped to KEGG IDs by the original authors. When these are converted to ChEBI IDs using the MetaboAnalyst conversion tool, and then back to KEGG IDs again, 251 compounds have identical identifiers. The remaining 16 compounds include 15 compounds that could not be mapped from KEGG to ChEBI, and one compound that was originally Catechol (C00090) and was mapped from ChEBI to KEGG to a different entry but for the same compound (Catechol - C15571). In summary, 94% of compounds in the Labbé dataset were back-converted to their original KEGG IDs, with just one mismatch (although to the same compound name) and 5% of compounds which could not map from KEGG →ChEBI originally.

In the discussion we have the following:

L620-631: "A further important consideration for pathway database evaluation is the type of compound identifiers used in the pathway. KEGG and BioCyc use database-specific identifiers, whereas Reactome uses ChEBI identifiers. ***It is necessary to convert the identifiers present in a metabolomics dataset to their database-specific equivalent, which often results in loss of information*** *as not all identifiers will necessarily map directly to a database compound or be annotated to a pathway [28]. For example, in the Stevens et al. dataset, over 900 compounds were assigned to Metabolon identifiers, but less than half of these compounds could be mapped to KEGG identifiers.* ***Another characteristic of metabolomics (and in particular lipidomics) is the discrepancy between the chemical precision of identification between the pathway databases and the dataset****. For instance, in databases classes of lipids are often gathered into a single element (e.g. "a triglyceride") while lipidomics allows more in-depth annotation (e.g. "TG 16/18/18")."*

We believe the current text highlights important issues associated with pathway compound ID conversion, including those mentioned by the reviewer. We appreciate this is a valid concern, but believe that to go into this issue in more detail would obscure the main message of the paper.

L567 – metabolite misidentification
We thank the reviewer for suggesting this change and we have re-named section 3 of the methods to "Metabolite misidentification".

Results

L169 – NMR is not relevant in this study, as none of the studies chosen have used it, so please remove it.
While we acknowledge that none of our example datasets use NMR, we still believe it is beneficial to discuss the importance of the background set size (essentially metabolite coverage) in ORA, which is often a result of the analytical platform used. We believe NMR is an important example of a platform where metabolite coverage is limited and have thus left it in. We hope this point will encourage readers to think carefully about the experimental design they have used and whether the analytical platform will provide sufficient coverage to carry out a reliable pathway analysis.

L293 – if the authors want to mention MSI levels 2-4, then they need to explain what these are, as the readers might not know…
We agree with the reviewer that not all readers may be familiar with MSI levels, and as such have elaborated on this in the text:

L411-418: "Consequently, a large proportion of compounds in untargeted metabolomics assays are expected to have a degree of uncertainty in their identification, ranging from Metabolomics Standards Initiative (MSI) confidence levels 2-4 [21]. These levels refer to the minimum reporting criteria for metabolite identification proposed by the MSI, in which a level 1 identified compound is one that has been identified using an authentic chemical standard, as opposed to levels 2-4, which range from a compound putatively identified based on physicochemical and/or spectral similarities to compounds in a spectral library (level 2), to an unknown compound (level 4)."

Fig5 – A and B figures are actually quite similar. So it might be worth mentioning in the text that the misidentification is probably from molecular formula to metabolite and not so much from mass to molecular formula. Some words on the similarly / differences between these two graphs are worth mentioning.

We thank the reviewer for highlighting the similarity between the two subfigures and have therefore decided to combine them into one, in order to highlight similarities and differences. This has replaced the original figure 5.

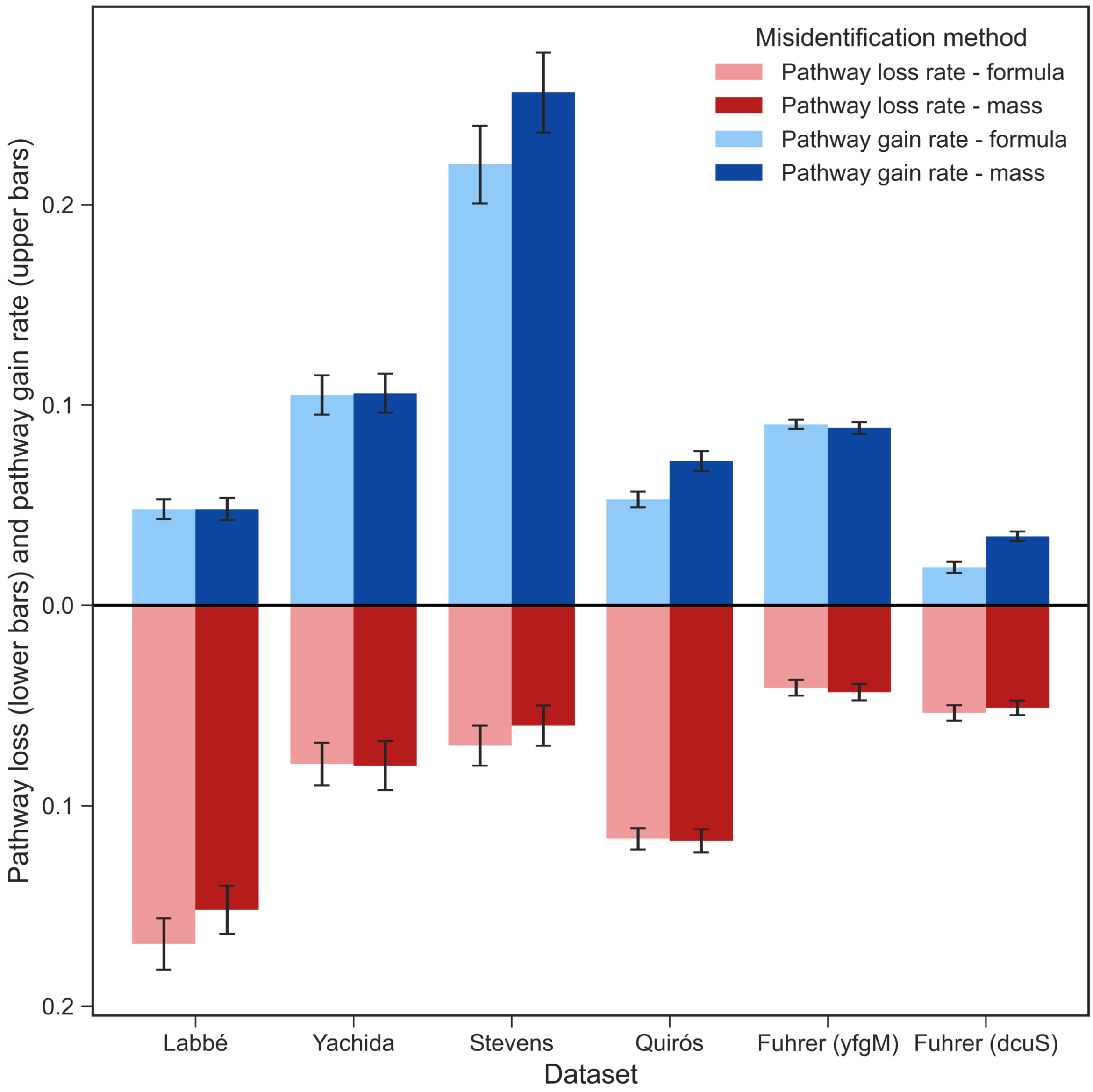

We have added the referee's excellent point to our discussion of this figure in the main text (L443-445).

L334 - 349 – one needs to be careful with these type of statements. Reversed phase is used in combination with ion pairing for detecting polar metabolites, of a similar nature to the ones that are detected by HILIC. HILIC can also detect a lot of apolar metabolites, because it can act in a mixed mode type of chromatography. In addition GC-MS with a prior derivatisation step in the sample preparation has been used a lot for detecting polar metabolites! So being that there is a lot of variety in analytical and sample prep methods for metabolomics, this whole section should be rephrased and adapted.
The authors should stick to polarity of compounds to make their point, irrespective of the technique used, as clearly the reality is not this simple, as it does not only depend on chromatography!! in fact one of the datasets does not use chromatography but capillary electrophoresis!!
Then none of the datasets aimed at lipid metabolism, this would then lead to completely different result. So this whole section is very circumstantial and simply not informative.

We agree with the reviewer that there are nuances to each type of chromatography and that it may be best to focus on the polarity of compounds detected. We understand that to some readers and specifically those experienced in metabolomics, the point we are making about assay chemical bias may seem trivial, but to others such as early career researchers or those new to metabolomics this may not be so obvious.

We therefore opted to keep this section of the results but have added some new figures and analysis which we believe makes the point more clearly. We have taken one of the datasets, Stevens et al., and plotted the compounds detectable using each of the four assays from their study onto the KEGG metabolic network. We have also compared the pathways accessible using the compounds detected by each assay and the overlap between them. Please see section 5 of the results "The polarity of compounds in a metabolomics experiment influences the pathways discoverable using ORA".

Discussion
L395 – mis-identification is abundant in all analytical platforms!
We agree with the reviewer that misidentification is no doubt abundant. However, we do want to make the point that it is both important, but variable according to the analytical method employed. We have adjusted the text slightly to indicate that this applies to most studies: L593 "most studies will contain at least some misidentified compounds"

L397 – not relevant to mention NMR as it was not used in this study. To add to this: maybe NMR provides less coverage but maybe better identification…?
We refer to our response to the reviewer's earlier comment about NMR.

Reviewer 3

Reviewer #3: Wieder and colleagues performed an interesting study on the application of ORA to metabolomics data. The paper is well-written and proposes, for the first time, the guidelines to perform ORA analysis in metabolomics. I especially enjoyed reading the pathway comparison part, it is a nice addition to the paper. However, some of the observations or conclusions were somewhat trivial to me. Still, I find the paper suitable for publication and I suggest the following changes to improve the paper:

- The authors state: "To perform ORA, three essential inputs are required: a collection of pathways (or custom metabolite sets), a list of metabolites of interest, and a background or reference set." By definition, all annotatable metabolites in untargeted metabolomics are all those in the collection of pathways. How do all annotatable metabolites and all metabolites in the pathway differ?

We thank the reviewer for their comment. We believe there may be confusion over the word "annotation" which in metabolomics usually means assignment of a tentative molecular identity to a feature (e.g. a peak) in the dataset. By "all annotatable metabolites", we refer to all metabolites in a particular dataset which can be annotated to a compound name or ID. An example in our work is the Stevens dataset, which had 949 metabolites annotated to common names based on their m/z ratios and retention times using the Metabolon internal compound library. When using KEGG, the background set will be smaller as not all these compounds map to KEGG pathways. "All metabolites in the pathway database" refers to the

set of metabolites which form the pathways in the database e.g. KEGG human pathways, which contain over 3,300 compounds that will not all necessarily be present or annotated in a dataset. We have carefully checked the manuscript to ensure that we only use the word annotation in the sense defined above and hope that this clarifies this point.

- Pg 12, section "increasing the number...". It is not needed to do all that to demonstrate this trivial aspect. It is expected. The authors could perform a similar approach but instead of considering all pathways, considering only those pathways that have at least 2 (or 3 if data allows it) DA, and then randomly add new DA to the pathways to see how the overall ranking fluctuates. Otherwise, adding DA by p-value is arbitrary and, considering the nature of untargeted metabolomics data, these observations are expected.
We agree with the reviewer that this aspect of the paper may seem trivial to some readers. However we believe many may not fully appreciate the sensitivity of ORA to the number of differentially abundant metabolites. We also believe that the figure we have produced to demonstrate this trend (Fig 3) may inspire readers to produce a similar plot to explore their dataset and view the effects of potential cut-off thresholds for the list of metabolites of interest. Finally, as the list of metabolites of interest is a key parameter of ORA and greatly affects the results, we have made the decision to keep this section as is in the manuscript.

We also thank the reviewer for the suggested analysis they have proposed. While we do not question the significance or validity of the proposed approach, we believe that the current analysis performed and shown in Fig. 3 addresses the question of p-value cut-offs most clearly. It aligns to the common practice of experimenting with different p-value cut-offs to determine the effect on the number of significant pathways. We also believe the way we have presented the data would be more easily interpretable than the suggested approach, which would include non-DA metabolites and relies on comparing rankings.

- "Pathway sets can be obtained freely from several databases..". . KEGG is partially commercial so should not be included. For BioCyc, I would like to know how the authors obtained that information as I believe it's partially commercial. MetExplore uses others databases so it should be removed as well. Is Ingenuity still on business?
We thank the reviewer for highlighting these points. We have removed the word "freely" and the reference to MetExplore. To the best of our knowledge, Ingenuity Pathway analysis by Qiagen is still used by researchers in both the metabolomics and transcriptomics communities, and therefore we wish to keep this reference in the text.

L156-158: "Pathway sets can be obtained from several databases, for example, the Kyoto Encyclopaedia of Genes and Genomes (KEGG) [15], Reactome [16], and BioCyc [17] databases, or commercial counterparts such as the Ingenuity Pathway Analysis (IPA) database [19]."

BioCyc provides a free academic license for those at academic/government institutions wishing to use their database for research purposes (https://biocyc.org/download-bundle.shtml). This provides access to all the pathway data in their databases which can be downloaded as flat files (.tsv) or filtered and exported using the SmartTables function.

- Pg 26: "Suggested recommendations...". The paper discusses the ambiguity of the

composition of the background set in untargeted metabolomics, but the recommendations are not clear on how this background set should be built in untargeted metabolomics. It would be worthwhile to break down the first recommendation into untargeted and targeted.

We thank the reviewer for highlighting this source of ambiguity in our suggested recommendations. We think that defining the "ideal" background set remains an open question for untargeted studies. Therefore, to make our recommendations as practical as possible, we have added the following:

L757-759: "1. Specify a realistic background set based on the analytical platform used in the experiment. A conservative yet practical approach is to use all the metabolites that have been identified in the assay."

We believe the use of all identified metabolites is the safest approach to defining a background set that will avoid the emergence of false positive pathways when the true background set remains unknown. We have added the following to the discussion to highlight the ambiguity surrounding the true nature of what an assay-specific background set should contain:

L563-576: "Defining the ideal assay-specific background set for a particular dataset remains an area for further study. The approach used in this work was to use all identified compounds, which although conservative, is the safest approach minimising the number of false-positive pathways. The ideal assay-specific background set may be broader and is subject to considerations such as the compounds present in the spectral library used for identification, those above the detection limit and well quantified for the instrument used, and those expected to be present in the organism and sample source investigated."

- Discussion, how using topology-based or FCS do/could naturally overcome some of the ORA limitations, or introduce different biases. Could the recommendations be instead: do not use ORA, but FCS/Topology-based? What could the limitations of FCS/Topology-based in untargeted metabolomics be? I believe a brief discussion about this is necessary.

We thank the reviewer for this highly relevant suggestion for the recommendations. Although we agree with the reviewer that this would be a desirable discussion point, we feel that as we have not employed FCS and topology-based methods in this work, that we lack sufficient evidence to make a sound recommendation.

While we can speculate the various benefits and limitations of FCS/topology based methods applied to metabolomics data (e.g. a disadvantage of topology would be that the often poor coverage of the metabolic network would make calculating topology-based statistics less reliable), there has been little work done to investigate this. We therefore leave this to future work.

- Pg 7 lines 139; "consisting of all compounds annotated to at least one KEGG pathway", could you define this better?

We thank the reviewer for highlighting that the clarity of this sentence could be improved. We have reworded this section to improve the clarity and highlight that the non-assay specific background set we used consisted of all unique compounds present in the organism-specific KEGG pathway set (i.e. for human datasets, this would be all 3373 compounds present in the KEGG human pathway database).

L214-217: "We investigated the effect of using a nonspecific background set, consisting of all unique compounds present in the KEGG organism-specific pathway set, compared to an assay-specific background set, consisting only of compounds identified and present in the abundance matrix of each dataset."

We believe the clarification of the word 'annotation' discussed earlier will also aid understanding of this aspect.

Minor:

We thank the reviewer for highlighting the following minor points and have rectified them accordingly.

- Change: Firstly -> first, secondly -> second

The correction has been made.

- Pg 5 line 95: p-value, P should be capitalized.

The correction has been made.

- Pg 14 line 225. "Pathway database is key" I suggest using a more informative sentence.

This has been changed to: "ORA results are influenced by pathway database choice, organism-specificity, and database updates".

- I did not find the supplementary materials.

We believe the supplementary materials are downloadable via a link at the end of the PDF compiled by the PLOS editorial system.
