# Supplementary material for: Pathway analysis in metabolomics: Recommendations for the use of over-representation analysis
Source: PLoS Comput Biol. 2021 Sep 7;17(9):e1009105. doi: 10.1371/journal.pcbi.1009105 (PMC8448349; doi:10.1371/journal.pcbi.1009105)
Supplement: Attachment — Submitted filename: Letter_to_editor_v2.docx [file pcbi.1009105.s003.docx]

Section of Bioinformatics

Division of Systems Medicine

Department of Metabolism, Digestion and Reproduction

Imperial College London

Office 131 Sir Alexander Fleming Building

Exhibition Road

South Kensington

London SW7 2AZ

Tel: +44 (0)20 7594 3160

[t.ebbels@imperial.ac.uk](mailto:t.ebbels@imperial.ac.uk)

<http://www.imperial.ac.uk/people/t.ebbels>

18 August 2021	**Dr. Tim Ebbels** BA PhD FRSC

Reader in Computational Bioinformatics

Dear Editor,

Thank you for your email and for the reviewers’ comments concerning our manuscript entitled “Pathway analysis in metabolomics: recommendations for the use of over-representation analysis”.

We sincerely appreciate your valuable feedback and are glad to hear that the reviewers have received our revised manuscript positively, with no further changes required. As per your recommendation, we have changed the title of the manuscript to “Pathway analysis in metabolomics: recommendations for the use of over-representation analysis”. We have resubmitted a copy of the manuscript (tracked changes copy, clean copy, and supplementary information) reflecting this change.

We look forward to hearing from you regarding our submission and to respond to any further questions and comments you may have.

Yours sincerely,

Timothy Ebbels

Imperial College London
